# Acute docosahexaenoic acid administration reduces binge-like ethanol consumption and palatable substances in C57BL/6J mice

**DOI:** 10.3389/fphar.2026.1891616

**Published:** 2026-08-24

**Authors:** Ainhoa Sánchez-Gil, Francisca Carvajal, Diana Cardona, Jose Manuel Lerma-Cabrera

**Affiliations:** 1 Department of Psychology, University of Almeria, Almeria, Spain; 2 Health Research Center, CEINSA, University of Almeria, Almeria, Spain; 3 Department of Nursing, Physiotherapy and Medicine, University of Almeria, Almeria, Spain

**Keywords:** binge-like drinking, DHA, drinking-in-the-dark, ethanol, palatable substances

## Abstract

**Introduction:**

Excessive alcohol consumption, particularly binge drinking, is associated with cognitive impairments, emotional dysregulation and an increased vulnerability to alcohol use disorders. Emerging evidence suggests that docosahexaenoic acid (DHA), an omega-3 polyunsaturated fatty acid, modulates neuroinflammatory processes and influences dopaminergic signaling involved in reward-related behaviors. However, its acute effect on binge-like consumption remains poorly understood. The present study investigated the effects of acute oral administration of DHA-rich fish oil on binge-like drinking of ethanol in male and female C57BL/6J mice, utilizing caloric and non-caloric reinforcers to evaluate behavioral specificity.

**Methods:**

Using the Drinking-in-the-Dark (DID) paradigm, voluntary intake of ethanol, sucrose and saccharin were evaluated following DHA administration. To control potential confounds, spontaneous locomotor activity and anxiety-like behavior were assessed.

**Results:**

Acute DHA administration significantly reduced binge-like consumption of ethanol in both sexes, with a parallel reduction observed in sucrose and saccharin intake. Importantly, DHA did not alter locomotor activity or anxiety-like behaviors.

**Conclusion:**

These findings suggest that DHA exerts a rapid and robust dampening effect on binge consumption of both ethanol and natural rewards, supporting a potential role of DHA in modulating reward-driven intake. Collectively, this study highlights DHA as a promising candidate modulator of neurobehavioral processes associated with excessive and non-homeostatic consumption.

## 1 Introduction

Excessive alcohol consumption represents a major global public health issue, acting as a significant risk factor for the development of multiple pathologies, including metabolic alterations, hepatic complications and cardiovascular diseases, among others (Spear, 2018; World Health Organization, 2024). Of particular concern is binge drinking, the consumption of large amounts of alcohol over a short period of time, which is increasingly prevalent among adolescents and young adults, and occurs more frequently in these populations than in older adults (National Institute on Alcoholism and Alcohol Abuse, 2004; Addolorato et al., 2018). This pattern of ethanol intake has been linked to poor inhibitory control, increased impulsivity, cognitive flexibility deficits, and emotional dysregulation (Spear, 2018; Valencia Martín et al., 2020; Le et al., 2022). Furthermore, frequent binge episodes are strongly associated with an increased vulnerability to developing alcohol use disorder (AUD) (Addolorato et al., 2018). Consistent with these clinical observations, preclinical research indicates that binge-like ethanol exposure triggers neuroplastic changes that promote a transition towards a dependence-like state (Lowery-Gionta et al., 2012; Cox et al., 2013). AUD is characterized by complex neurochemical adaptations involving multiple neurotransmitters and neuromodulatory system, including dopaminergic, glutamatergic, GABAergic, opioid and endocannabinoid signaling, as well as neuroimmune processes that collectively contribute to reinforcement, craving and relapse vulnerability (Koob and Volkow, 2016; Prajapati et al., 2020). Therefore, elucidating the neurochemical pathways that modulate binge drinking, as this knowledge could provide valuable insights into pharmacotherapies to reduce this risky pattern of ethanol intake and prevent the progression to AUD. In this regard, a growing body of research suggests that repeated binge episodes act as a recurrent neurotoxic insult that induces neuroinflammatory responses characterized by microglial activation and increased pro-inflammatory cytokine release, particularly within mesolimbic regions involved in reward processing and motivation (He and Crews, 2008; Marshall et al., 2016). Concomitantly, excessive alcohol consumption promotes neuroadaptations in the dopaminergic circuit, including altered dopamine release dynamics and receptor downregulation in reward-related areas (Barrios et al., 2025). Neuroinflammation and dopaminergic dysregulation should not be considered independent consequences of binge drinking, but rather as closely interconnected processes. While inflammatory mediators can disrupt the dopamine system, altered dopaminergic tone can also regulate neuroimmune activity through dopamine receptors expressed by glial and immune cells (Kohno et al., 2019; Ye et al., 2025). This bidirectional relationship may facilitate the shift from controlled to compulsive consumption, highlighting the need to identify new pharmacological strategies that target these integrated systems at early stages of the addiction cycle.

In this context, nutraceutical interventions have emerged as promising candidates for the treatment of neuropsychiatric disorder, including substance use disorders (Sarris et al., 2022). Among these, omega-3 polyunsaturated fatty acids (n-3 PUFAs) have demonstrated effectiveness in mitigating neuroinflammation-related mental disorders and substance abuse (Carvajal et al., 2023; Peng et al., 2021). Eicosapentaenoic (EPA) and docosahexaenoic acid (DHA) are pivotal n-3 PUFAs that can effectively cross the blood-brain barrier, thereby exerting central effects (Freund Levi et al., 2014). In consideration of alcohol consumption, it has been established that dietary supplementation with these n-3 PUFAs has the capacity to modulate ethanol sensitivity and consumption in a genotype-specific manner. For instance, a supplemented diet with long-chain n-3 increased alcohol preference in C57BL/6J mice but not in DBA/2J mice, whereas alcohol sensitivity was only intensified in DBA/2J mice (Wolstenholme et al., 2018). In contrast, Shi et al. (2019) reported that n-3 PUFAs did not reduce alcohol (8% *v/v*) liquid diet consumption but effectively prevented relapse by alleviating withdrawal and attenuating chronic alcohol-induced synaptic remodelling within the nucleus accumbens. Conversely, a DHA-rich diet leads to a substantial decrease in ethanol consumption in alcohol-preferring (P) rats (Le-Niculescu et al., 2011). This divergence underscores the importance of lipid profiles in intervention approaches. Furthermore, DHA is the most abundant n-3 PUFAs within the brain structural lipids, playing an essential role as a precursor for specialized pro-resolving mediators with potent anti-inflammatory properties (Dyall, 2015; Powell et al., 2021). Notably, upper-moderate but non-toxic levels of ethanol exposure has been shown to diminish brain DHA levels, consequently compromising the endogenous anti-inflammatory capacity (Schreiber et al., 2021). The findings presented herein suggest that DHA may preferentially modulate the behavioral processes underlying reinforcement and alcohol consumption.

Although DHA is commonly studied in the context of chronic dietary supplementation, several observations suggest that its acute effects are biologically plausible. DHA can cross the blood-brain barrier (Freund Levi et al., 2014) and rapidly modify biophysical properties of membranes, which influence receptor function and intracellular signaling. Experimental evidence indicates that membrane polyunsaturation regulates dopamine D2 receptor ligand binding and signaling efficacy (Jobin et al., 2023), while DHA-derived lipid mediators can rapidly modulate neuroimmune responses and inflammatory signaling pathways (Serhan, 2014). Because binge-like ethanol drinking is strongly dependent on transient changes in mesolimbic reward processing and motivational state, it is possible that a single DHA administration could influence consummatory behavior without requiring long-term structural adaptations. However, this possibility has received little experimental attention, representing an important gap in the literature. Therefore, the present study investigated the effects of acute oral administration of DHA-rich fish oil on binge-like drinking of ethanol, as well as caloric (sucrose) and non-caloric (saccharin) reinforcers, in both male and female mice using the DID paradigm. Additionally, we evaluated whether DHA administration affects locomotor activity or modulates anxiety-like behavior, providing a comprehensive evaluation of its neurobehavioral effects and its potential as a targeted intervention for addictive and compulsive behaviors.

## 2 Materials and methods

### 2.1 Animals

Male and female C57BL/6J mice (Janvier Labs, France) aged 5–7 weeks at the beginning of the experiments were used. The animals were individually housed in plastic cages and allowed to acclimate to their environment for at least 1 week before the start of the experiment. They had *ad libitum* access to food and water, except as noted. The colony room was maintained at approximately 22 °C with a 12:12 h light-dark cycle (lights off at 08:00 a.m.). All experiments comply with the ARRIVE guidelines and were carried out in accordance with Spanish Royal Decree 53/2013 and the European Community Directive 2010/63/EU on animal protection and experimentation.

### 2.2 Drug administration

DHA-triglyceride fish oil (Brudy Technology, Barcelona) containing 70% of DHA was administered via gavage (intragastric administration, i.g.) at a volume of 1 mL/100 g prior to the behavioral evaluation, corresponding to a DHA dose of 6,300 mg/kg. The vehicle (sunflower oil), which did not contain any omega-3 fatty acids, was administered in the same volume as the DHA solution.

During consecutive weekly DID sessions, a counterbalanced 2 × 2 Latin-square design was employed. Animals received both treatments (vehicle and DHA) in a randomized and counterbalanced order across two consecutives DID cycles separated by 1 week, such that each animal served as its own control for treatment comparisons.

### 2.3 Behavioral assessment

#### 2.3.1 Drinking in the dark (DID) procedure

**FIGURE 1:**
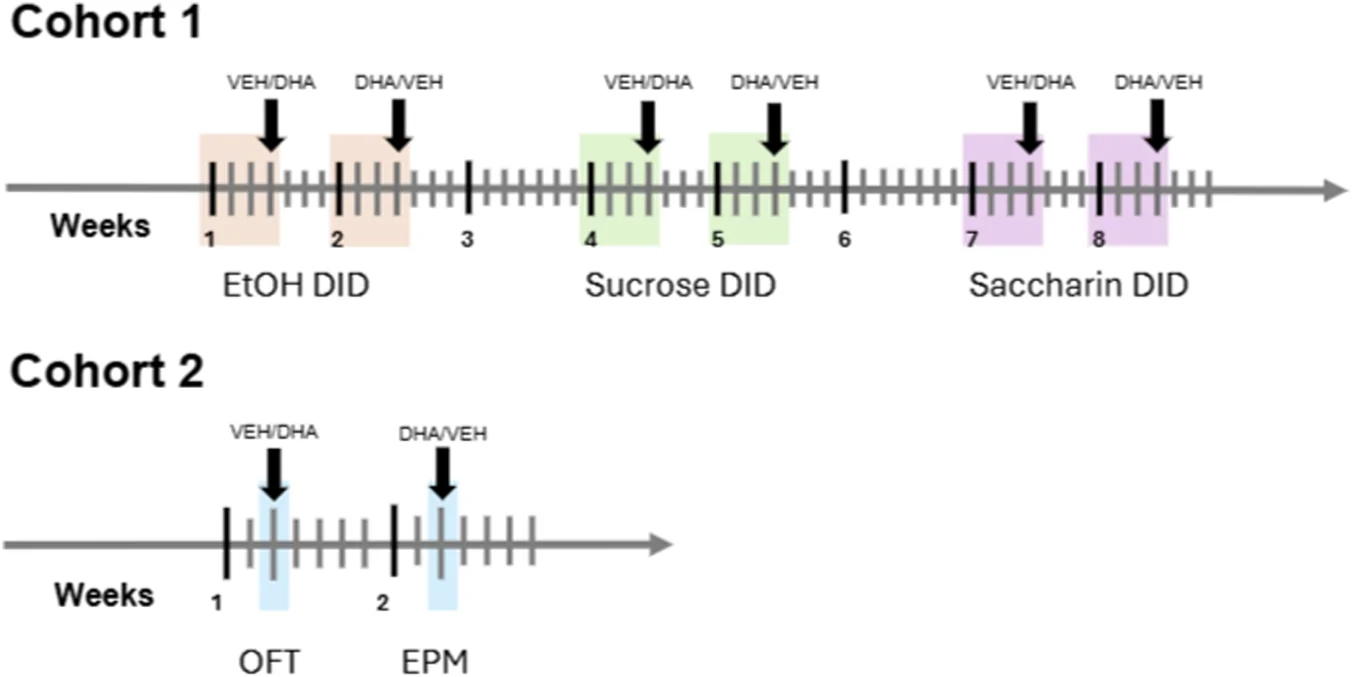
Schematic timeline for the experimental design. The study comprised two independent cohorts of mice. Cohort 1 underwent sequential Drinking-in-the-Dark (DID) procedures for ethanol, sucrose, and saccharin. For each substance, a 2 × 2 counterbalanced within-subject crossover design was employed over two consecutive cycles of DID to ensure that every animal received both treatments (Vehicle or DHA) on the testing day. Cohort 2 performed the Open Field Test (OFT) in week 1 and the Elevated Plus Maze (EPM) in week 2. Treatment assignments (Vehicle vs. DHA) were completely reversed between the two testing weeks following a 1-week rest period.

Mice were trained and tested using a standard 4-day DID procedure (Thiele et al., 2014). From days 1–3, 3 h into the dark period, the water bottle was replaced with one containing the corresponding solution for that experimental stage: 20% (v/v) ethanol, 3% (w/v) sucrose, or 0.15% (w/v) saccharin. Daily drinking access was limited to 2 h. On the fourth day, animals (n = 12; 6 males and 6 females) were administered, either DHA-rich fish oil or vehicle i. g. 90 min before the test, and then they were given access to the corresponding solution for 4 h. Fluid consumption was recorded at the end of each session, with an additional intermediate measurement taken after 2 h on the test day. Each 4-day DID was followed by a week before the next solution was introduced sequentially using the same cohort of animals (Figure 1).

To control fluid spillage, an empty cage set up with identical bottles was included, and any volume lost due to dripping was subtracted from the total consumption. Fluid consumption and body weight measures were recorded in each session. The amount of ethanol consumed was calculated as g of ethanol per kg of body weight (g/kg).

#### 2.3.2 Open field

To assesses locomotor activity, mice (n = 32; 16 males and 16 females) were evaluated in open-field chambers (42 cm^2^ × 42 cm^2^) equipped with photobeams (Versamax Animal Locomotor Activity, Madrid, Spain). The procedure used was adapted from Seibenhener and Wooten (2015). Before testing, mice were transported to the locomotor testing room in their home cages and allowed to habituate for 30 min. Following habituation, mice received their first assigned i. g administration of either DHA-rich fish oil or vehicle and were individually placed in the center of the activity chamber 2 h later. Locomotor activity was recorded for 10 min using the integrated Versamax software. The floor of the locomotor activity chamber was cleaned with 70% ethanol after each session. The behavioral measures recorded included total distance travelled, total movement time and time spent in the center of the activity chamber.

#### 2.3.3 Elevated plus maze

The same cohort of mice used in the open field test was evaluated in the elevated plus maze (EPM) to assess anxiety-like behavior 1 week later, utilizing a crossover and counterbalanced design (Figure 1). The apparatus consisted of two open arms (30 cm × 5 cm) and two enclosed arms (30 cm × 15 cm × 5 cm), extending from a central square (5 cm × 5 cm) and elevated 50 cm above the floor (Cibertec, S.A., Madrid, Spain). Following the protocol described by Alcaraz-Iborra et al. (2017), mice were acclimated to the experimental room for 30 min on two consecutive days prior to testing. On test day, mice were administered i. g. with their respective alternate treatment (DHA-rich oil or vehicle). Two hours later, each mouse was placed in the center of the maze facing an open arm, and its behavior was recorded for 5 min using an overhead camera and OBS Studio software (v27.2.3; OBS Project). The percentage of time spent in open and closed arms, as well as the total number of entries were quantified. Additionally, an anxiety index was calculated as follows: anxiety index = 1 – [(open arm time/300 s) + (open arm entry/total entry)]/2. Values for the Anxiety Index range from 0 to 1, where values closer to 1 indicate increased anxiety-like behavior.

Finally, specific ethological indicators, such as head dips and stretched-attend postures, were recorded to assess risk-taking and exploratory behavior. Also, the number of closed-arm entries was also recorded as an index of general activity, allowing the interpretation of anxiety-related measures independently of potential alterations in locomotion.

### 2.4 Statistical analysis

For the drinking experiments, mixed repeated-measures ANOVAs were performed with treatment (DHA vs. vehicle) as a whiting-subject factor and sex (male vs. female) as between-subjects factors. For locomotor activity and anxiety-related measures, two-way ANOVA with treatment (DHA vs. vehicle) and sex (male vs. female) as between-subjects factors, unless otherwise noted. Repeated measures ANOVA was used to compare open field test variables across two 5-min intervals. *Post hoc* comparisons were planned using Tukey’s test where appropriate. Statistical significance was set at p < 0.05. All values are reported as mean ± standard error of the mean (SEM). The analyses were performed using Statistica software (StatSoft, Inc.) version 8.0, and graphs were generated using GraphPad Prism (GraphPad Software) version 8.

## 3 Results

### 3.1 Experiment 1: effect of DHA on ethanol, sucrose and saccharin binge-like drinking in a DID procedure

**FIGURE 2:**
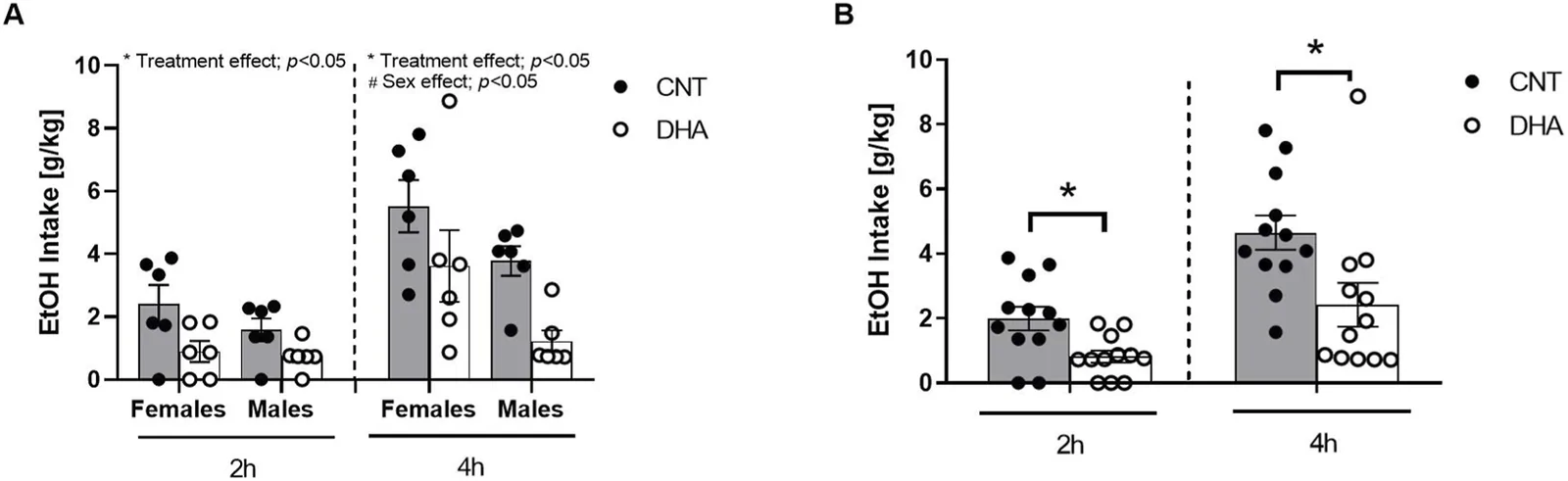
Binge-like ethanol (20%) consumption during the 2-h and 4-h testing in the DID procedure. **(A)** Ethanol intake in male and female mice treated with vehicle (CNT) or DHA. **(B)** Data collapsed across sex to illustrate the significant main effect of DHA treatment. All values are represented as means ± SEM with individual data points. Statistical analyses revealed significant main effects of treatment (*p < 0.05) and sex (#*p* < 0.05), but no significant Sex × Treatment interaction.

Figure 2 shows 20% ethanol intake (g/kg) during the 2 h and 4 h test day. On the test day, the analysis performed on the fourth hour of testing revealed that the female group consumed significantly more ethanol than the male group (F (1, 10) = 8.211; p = 0.016). This sex difference was not evident during the second hour of testing (F (1,10) = 1.351; p = 0.272). Notably, the pretreatment with DHA significantly reduced ethanol consumption (20% w/v) at both the 2-h (F(1,10) = 9.209; p = 0.012; Hedges’ g = 0.81, 95% CI [0.17, 1.42]) and the 4-h testing (F(1,10) = 7.678; p = 0.019; Hedges’ g = 0.74, 95% CI [0.11, 1.34]). No significant sex × treatment interaction was observed (p > 0.05).

**FIGURE 3:**
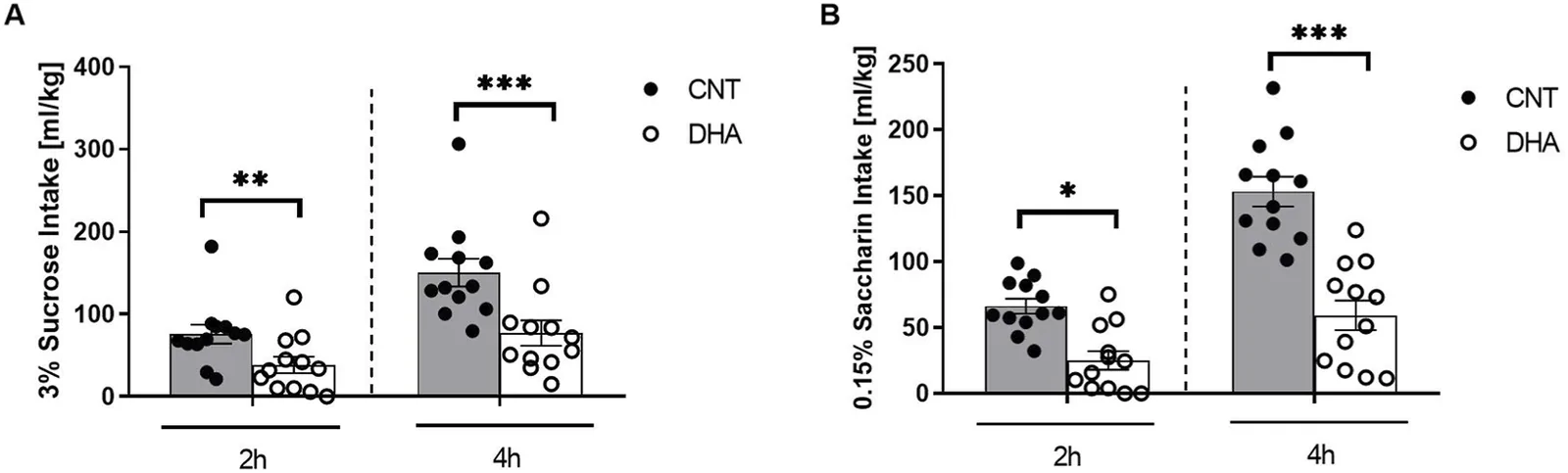
Consumption (mL/kg) of sucrose (3%) **(A)** and saccharin (0.15%) **(B)** during the 2-h and 4-h testing in the DID procedure. All values are represented as means ± SEM with individual data points. *p < 0.05, **p < 0.01, ***p < 0.001 for treatment differences.

The effect of pretreatment with DHA on the binge-like consumption of other reinforcing solutions, sucrose (caloric) and saccharin (non-caloric) are shown in Figure 3. Repeated measures ANOVA showed that DHA treatment resulted in a significant reduction of 30% sucrose intake at both 2 h (F (1,10) = 11.572; p = 0.006; Hedges’ g = 0.91, 95% CI [0.24, 1.55]) and 4 h (F (1,10) = 22.307; p = 0.0001; Hedges’ g = 1.26, 95% CI [0.49, 2.00]). No other effects (e.g., sex or interaction) were statistically significant.

Similarly, the consumption of 0.15% saccharin solution was significantly attenuated by DHA pretreatment at the second and the fourth hours of testing, as confirmed by a two-way ANOVA [Treatment: 2-h test (F(1,10) = 15.654; p = 0.027; Hedges’ g = 1.05, 95% CI [0.35, 1.73]) and 4-h test (F(1, 10) = 40.751; p = 0.0008; Hedges’ g = 1.70, 95% CI [0.79, 2.59])]. No other effects (e.g., sex or interaction) reached statistical significance.

### 3.2 Experiment 2: effect of DHA on locomotor activity

**FIGURE 4:**
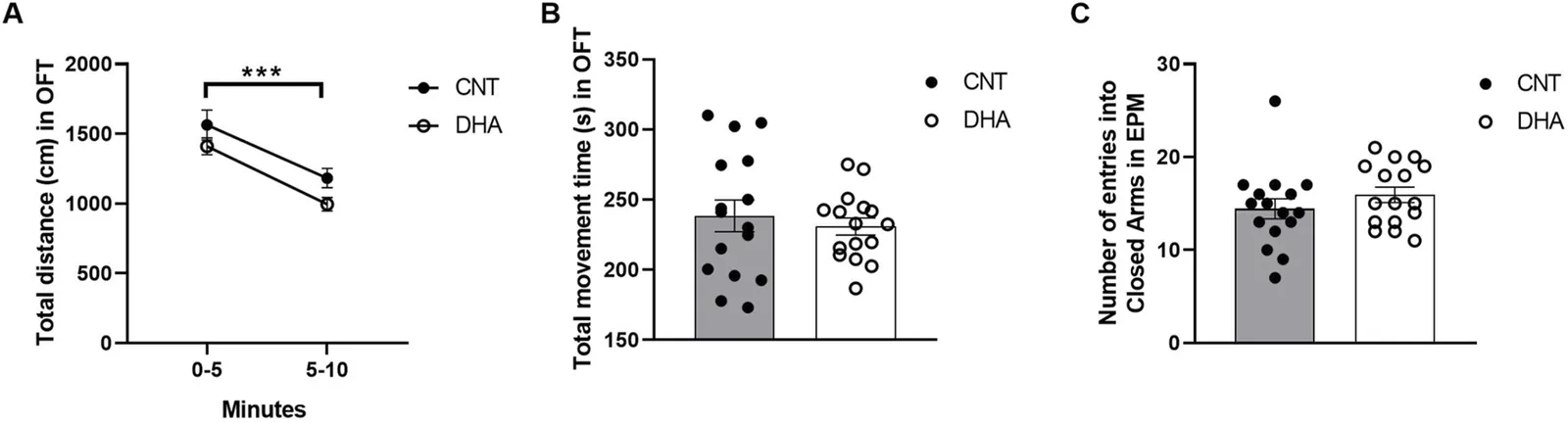
Locomotor and exploratory activity following DHA administration. **(A)** Total distance traveled (cm) in the open field test (OFT) across 5-min intervals (0–5 min and 5–10 min). **(B)** Total movement time (s) during the 10-min OFT. **(C)** Number of closed-arm entries in the elevated plus maze test (EPM). All values are represented as means ± SEM with individual data points; ***p < 0.001 for time bins differences.

To assess the effect of i. g. administration of DHA-rich oil on locomotor activity, animals were tested for 10 min in the open-field test. A repeated-measures ANOVA was performed to analyze the effects of time, DHA treatment, and sex across two 5-min intervals. There was a significant main effect of time (F(1, 28) = 83.722; p < 0.0001), as all animals significantly decreased their locomotion from the first to the second interval, indicating a normal habituation pattern (Figure 4A). However, not significant differences were found in total distance traveled [sex (F(1, 28) = 0.018; p = 0.893), treatment (F(1, 28) = 2.890; p = 0.100) and interaction (F(1, 28) = 0.001; p = 0.983)]. Similarly, total movement time (Figure 4B) was not affected by sex (F(1, 28) = 0.614; p = 0.439), treatment (F(1, 28) = 0.326; p = 0.572) or the interaction (F(1, 28) = 0.038; p = 0.846).

The number of entries into closed arms was also assessed as an internal control for locomotor activity in the EPM (see Figure 4C). A two-way ANOVA (sex × treatment) showed no statistical differences in sex (F(1, 28) = 0.310; p = 0.582), treatment (F(1, 28) = 1.241; p = 0.274) or the sex × treatment interaction (F(1, 28) = 1.939; p = 0.174). Consistent with the OFT data, the absence of differences in closed-arm entries further indicates that acute DHA administration did not impair general locomotor performance.

### 3.3 Experiment 3: effect of DHA on anxiety-like behavior

**FIGURE 5:**
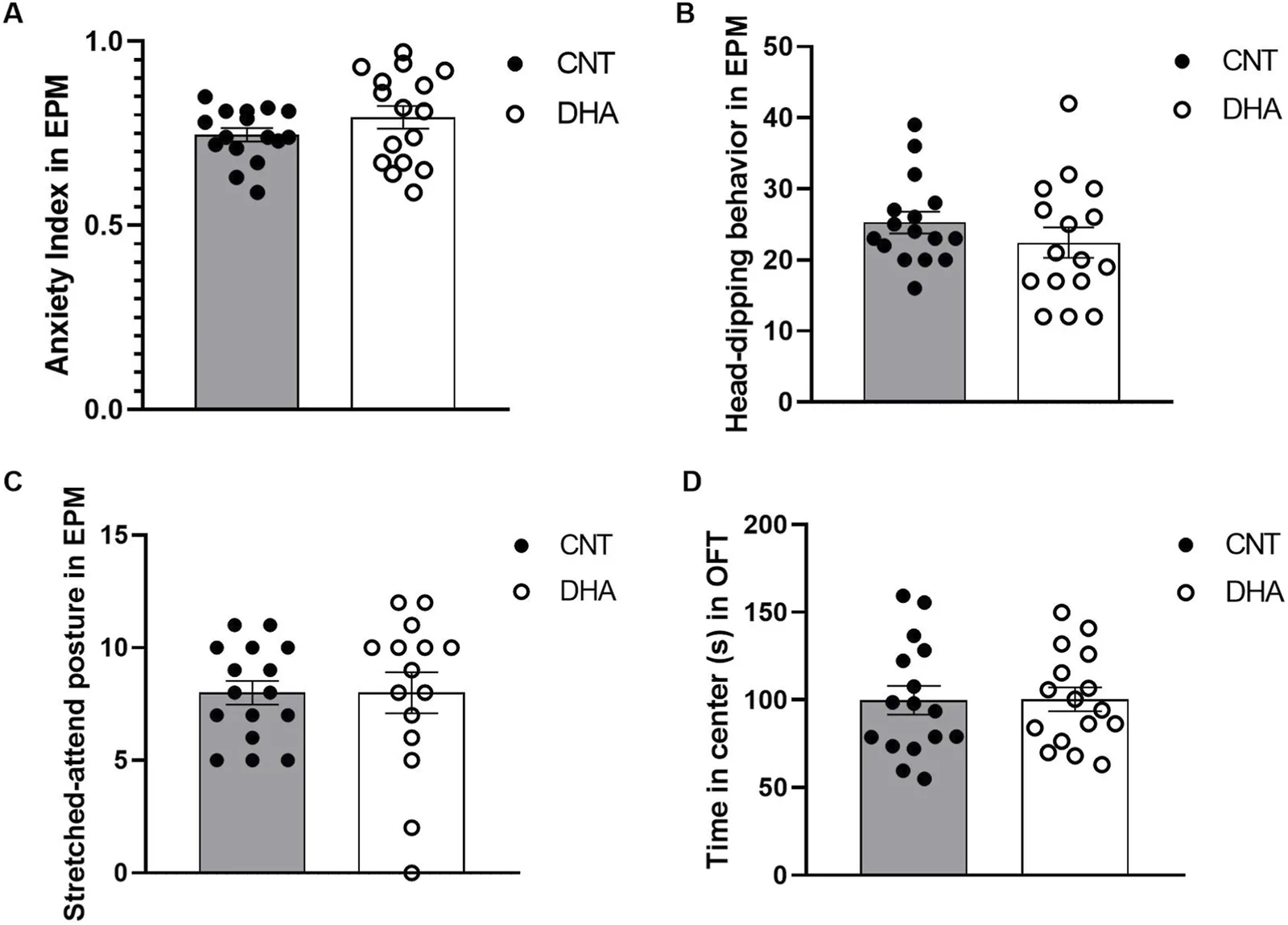
Anxiety-like behaviors following acute DHA administration. **(A)** Anxiety index and ethological measures including **(B)** head-dipping behavior and **(C)** stretched-attend postures assessed during the 5-min EPM test. **(D)** Time spent (s) in the center zone during the 10-min open field test (OFT). All values are represented as means ± SEM with individual data points.

Figures 5A–C shows results from the elevated plus maze, used to evaluate anxiety-like behavior. A two-way ANOVA (sex × treatment) revealed no significant differences in the percentage of time spent in open or closed arms, nor in the total number of entries (Table 1). Similarly, the anxiety index was not affected by sex (F(1, 28) = 0.223; p = 0.640), treatment (F(1, 28) = 1.646; p = 0.210) or the interaction (F(1, 28) = 0.164; p = 0.688). Furthermore, risk assessment and exploratory behavior, including the number of head-dips and stretched-attend postures, showed not significant differences between groups [head-dips: sex (F(1, 28) = 0.602; p = 0.444), treatment (F(1, 28) = 1.120; p = 0.298) and interaction (F(1, 28) = 0.094; p = 0.762); stretched-attended postures: sex (F(1, 28) = 0.234; p = 0.632), treatment (F(1, 28) = 0.014; p = 0.904) and interaction (F(1, 28) = 0.059; p = 0.810)] between groups.

**TABLE 1:** Behavioral data obtained during elevated-plus maze test in control and DHA groups.

Treatment	% Time closed arms	% Time open arms	Number of closed arms entries	Number of open arm entries	Number of total entries
Control	38.64 ± 2.52	17.88 ± 1.70	14.44 ± 1.07	6.88 ± 0.49	21.31 ± 1.23
DHA	44.32 ± 2.42	13.60 ± 2.44	15.94 ± 0.83	6.50 ± 1.05	22.44 ± 1.10

A two-way ANOVA (sex × treatment) showed no significant differences. All data are represented as mean ± S.E.M.

Additionally, time spent in the center of the OFT remained unaltered by sex (F(1, 28) = 0.331; p = 0.569), treatment (F(1, 28) = 0.153; p = 0.698) or the interaction (F(1, 28) = 2.592; p = 0.118) (Figure 5D). Together, these findings indicate that basal exploratory anxiety was not modified by DHA treatment.

## 4 Discussion

The present study investigated the effect of acute DHA administration on the modulation of voluntary binge-like ethanol consumption. Our results demonstrated that DHA administration blunted binge-like ethanol intake in both male and female mice. Although female mice exhibited a higher baseline of ethanol consumption than males, consistent with established sexual dimorphism in alcohol drinking behavior (Middaugh et al., 1999; Tambour et al., 2008; Crabbe et al., 2009; Bendrath et al., 2025), the modulatory effect of DHA appeared to be comparable across sexes. Notably, recent studies found no sex-dependent differences in basal brain DHA levels between males and females under standard dietary conditions (Chen et al., 2020), which may partially account for the similar pharmacological responsiveness observed across both sexes in the present study. Nevertheless, sex-specific mechanisms governing reward-seeking cannot be excluded and should be addressed in future studies.

Furthermore, the observation that DHA reduced the consumption of both caloric (sucrose) and non-caloric (saccharin) palatable solutions is highly revealing. Given that these reinforcers differ in their metabolic value and pharmacological properties (Berridge and Robinson, 2016; Serafine et al., 2021), their common suppression of intake suggests that DHA acts on neurobiological mechanisms common to both natural and drug rewards, rather than a mechanism driven solely by metabolic feedback or ethanol-specific pharmacodynamics. However, since non-rewarding fluid was assessed, it remains unclear whether DHA selectively modulates reward-driven consummatory behavior or more generally reduces fluid intake. Crucially, this effect appears specific to consummatory behavior, as DHA did not alter locomotor activity or anxiety-like responses. This dissociation suggests that the neurobiological substrates of reinforcement might exhibit a higher sensitivity to acute DHA modulation than those governing basal affective states. Furthermore, the lack of anxiolytic effects in our paradigm, which contrasts with some chronic supplementation studies (Larrieu et al., 2014; Su et al., 2018), likely reflects the acute nature of the administration, which may be sufficient to dampen high-salience reward signaling without shifting the animal’s basal emotional tone. Together, these findings are consistent with the possibility that DHA modulates behavioral process underlying the consumption of both natural and drug rewards, independently of gross motor or affective impairments.

An emerging body of preclinical evidence supports the notion that dietary levels of n-3 PUFAs significantly influence the consumption of alcohol and other palatable substances. Converging evidence from preclinical studies indicates that alterations in n-3 PUFA availability, particularly during critical development periods, can modulate reward-related behaviors (Chalon, 2006; Ducrocq et al., 2020). For example, n-3 PUFA deficiency during prenatal and early development, which reduces brain DHA levels, has been shown to increase incentive motivation for sucrose in early adulthood (Auguste et al., 2018). Similarly, maternal diets characterized by an imbalanced n-3/n-6 ratio can modulate the motivational drive to obtain sucrose-containing solutions in the offspring, potentially via developmental changes in the midbrain dopaminergic system (Sakayori et al., 2020). In the context of alcohol consumption, available findings highlight the importance of specific fatty acid profiles. While DHA-rich diets have been reported to reduce voluntary alcohol consumption in alcohol-preferring P rats (Le-Niculescu et al., 2011), diets enriched in n-3 PUFAs but with higher EPA-to-DHA ratios have been associated with increased ethanol consumption in ethanol-preferring B6 mice (Wolstenholme et al., 2018). These preclinical observations align with clinical evidence showing reduced levels of n-3 PUFAs in heavy drinkers (Vatsalya et al., 2020) and identifying low plasma levels of DHA as a predictor of relapse in individuals with substance use disorders (Buydens-Branchey et al., 2009). Within this framework, the present findings extend previous work by demonstrating that even acute DHA administration during adulthood is sufficient to reduce binge-like consumption, although the underlying biological mechanisms driving this acute effect remain to be determinated. Nevertheless, differences in fatty acid composition, timing of exposure, and experimental paradigms across studies warrant caution when drawing direct comparisons. In particular, the present study employed a DHA-rich fish-oil formulation (>70% DHA) rather than purified DHA. Therefore, although DHA is the most likely mediator of the observed effects, contributions from other constituents of the formulation cannot be entirely excluded.

Converging evidence implicates the mesolimbic dopaminergic pathway, particularly projections from the ventral tegmental area to the nucleus accumbens, in the regulation of motivational processes and incentive salience underlying both natural and drug reward seeking (Koob and Volkow, 2016). In this context, n-3 PUFA status, and specifically DHA availability, emerges as an important modulator of dopaminergic function (Chalon, 2006; Ducrocq et al., 2020). Indeed, PUFAs play an important role in the development and maintenance of mesocorticolimbic dopaminergic projections, particularly within the prefrontal cortex and striatal regions (Healy-Stoffel and Levant, 2018). Experimental studies have shown that n-3 PUFA deficiency alters membrane lipid composition in these regions and is associated with impaired dopamine transmission and motivational deficits (Bondi et al., 2014; Ducrocq et al., 2020). Conversely, DHA enrichment of neuronal membranes has been reported to enhance ligand binding at the dopamine D2 receptor (Jobin et al., 2023), while n-3 PUFAs supplementation can increase dopamine transporter (DAT) and D2 receptor expression in models of dopaminergic dysfunction (Wu et al., 2021; Barroso-Hernández et al., 2022). Therefore, although the behavioral effect observed here may be discussed within a dopaminergic framework based on previous literature, the present data do not allow conclusion regarding the implication of specific dopamine-related mechanisms, and such interpretation should be considered hypothetical.

In parallel, increasing evidence indicates that neuroinflammatory processes contribute to the development and maintenance of alcohol use disorders and compulsive reward seeking (e.g., Koob and Volkow, 2016). Ethanol exposure, particularly under a binge-like pattern of intake, has been shown to induce rapid neuroinflammatory responses characterized by increased expression of pro-inflammatory cytokines and glial activation (Marshall et al., 2016; Crews et al., 2017). These neuroimmune changes can, in turn, modulate synaptic plasticity and dopaminergic transmission, thereby influencing the motivational states and reinforcing properties of ethanol (Crews et al., 2017). At the mechanistic level, activated microglia release cytokines, such as IL-1β, TNF-α, and IL-6, which can downregulate tyrosine hydroxylase expression, reduce dopamine release, and disrupt DA transporter trafficking (Yoshioka et al., 2022). Consistent with this interaction, animal models such as chronic unpredictable mild stress exhibit elevated levels of pro-inflammatory cytokines alongside anhedonic-like behaviors, such as reduced sucrose preference (Farooq et al., 2012), supporting a functional link between neuroimmune activation and reward deficits. Given that DHA is as a precursor of specialized pro-resolving mediators (e.g., resolvins, protectins, maresins) with potent anti-inflammatory and neuroprotective properties (Serhan, 2014; Ostrea et al., 2025), one might theorize a role for the modulation of neuroimmune environment. Notably, bidirectional interactions between neuroinflammatory pathways and dopaminergic function have been described, suggesting that changes in neuroimmune tone may influence reward processing at multiple levels (Crews et al., 2017; Channer et al., 2023). However, because our behavioral assessments were conducted shortly after a single, acute administration of DHA, attributing these rapid behavioral outcomes to an active resolution of neuroinflammation or specific resolvin pathways is highly speculative. Given the absence of direct neurochemical or inflammatory measurements in our study, these preliminary hypotheses must be interpreted with extreme caution and need to be explored in future studies utilizing pharmacological antagonists or genetic tools will be essential to definitively establish the necessity of D2 receptor signaling or specific resolvins pathways in these effects.

Acknowledging the scope of the present study, it is important to note that the observed effects of DHA were captured under an acute administration protocol. While this design was sufficient to demonstrate the immediate modulatory capacity of DHA on binge-like consumption, it does not account for the potential impact of chronic supplementation, which might yield different outcomes regarding basal anxiety or long-term neuroplasticity. Given that alcohol use disorders and the overconsumption of palatable foods are typically chronic conditions, future research is warranted to determine whether the suppressive effects of DHA are maintained, enhanced, or subject to tolerance over time. Furthermore, investigating whether long-term dietary enrichment leads to more stable structural changes in the mesolimbic circuitry compared to the acute effect observed here would be of high clinical relevance. Nonetheless, the fact that a single pretreatment was capable of significantly reducing the consumption of ethanol, sucrose and saccharin provides a compelling proof-of-concept for the use of DHA as a rapid-acting modulator of reward-driven consummatory behavior. However, given that ethanol, sucrose and saccharin were evaluated sequentially in the same cohort, cumulative confounds from prior DHA exposure, repeated gavages, aging, or the fixed testing sequence cannot be entirely excluded. Thus, these findings reflect reduced consumption across sequential DID procedures rather than independent suppression under completely naïve conditions. These findings provide a foundation for future studies aimed at determining the dose dependency, duration, neurobiological mechanisms, and translational relevance of this effect, which should be validated using independent cohorts and/or a counterbalanced reinforcer order.

## 5 Conclusion

In conclusion, the present findings provide preliminary preclinical evidence that acute administration of DHA-rich oil effectively reduces binge-like consumption of both ethanol and palatable rewards in both sexes across sequential testing procedures. While these results support a broader role for DHA-rich fish oil on excessive consummatory behavior rather than a selective action on alcohol-related behavior, the sequential design warrants caution, as it captures the effects of cumulative experimental procedures rather than independent states. Importantly, this suppression occurs independently of changes in basal anxiety or motor activity. Although these findings are compatible with an effect on mechanisms underlying reward-driven consumption, incorporating non-rewarding fluid controls in future studies will be necessary to establish the behavioral specificity of this effect. Ultimately, further studies integrating mechanistic, pharmacokinetic, and long-term behavioral approaches will be necessary to determine the translational relevance of these findings for alcohol use disorders and related conditions.

## Data Availability

The original contributions presented in the study are included in the article/supplementary material, further inquiries can be directed to the corresponding authors.
